# Dense carbon-nanotube coating scaffolds stimulate osteogenic differentiation of mesenchymal stem cells

**DOI:** 10.1371/journal.pone.0225589

**Published:** 2020-01-10

**Authors:** Hideki Mori, Yuko Ogura, Kenta Enomoto, Masayuki Hara, Gjertrud Maurstad, Bjørn Torger Stokke, Shinichi Kitamura

**Affiliations:** 1 Graduate School of Science, Osaka Prefecture University, Sakai, Osaka, Japan; 2 Graduate School of Life and Environmental Sciences, Osaka Prefecture University, Sakai, Osaka, Japan; 3 Biophysics and Medical Technology, Department of Physics, NTNU The Norwegian University of Science and Technology, Trondheim, Norway; 4 Center for Research and Development of Bioresources, Osaka Prefecture University, Sakai, Japan; Università degli Studi della Campania, ITALY

## Abstract

Carbon nanotubes (CNTs) have desirable mechanical properties for use as biomaterials in orthopedic and dental area such as bone- and tooth- substitutes. Here, we demonstrate that a glass surface densely coated with single-walled carbon nanotubes (SWNTs) stimulate the osteogenic differentiation of rat bone marrow mesenchymal stem cells (MSCs). MSCs incubated on SWNT- and multi-walled carbon nanotube (MWNT)-coated glass showed high activities of alkaline phosphatase that are markers for early stage osteogenic differentiation. Expression of *Bmp2*, *Runx2*, and *Alpl* of MSCs showed high level in the early stage for MSC incubation on SWNT- and MWNT-coated surfaces, but only the cells on the SWNT-coated glass showed high expression levels of *Bglap* (*Osteocalcin*). The cells on the SWNT-coated glass also contained the most calcium, and their calcium deposits had long needle-shaped crystals. SWNT coating at high density could be part of a new scaffold for bone regeneration.

## Introduction

Today, carbon nanotubes (CNTs) are just beginning to be used in various industries; including sporting goods manufacturing, the automotive industry and electronics, because they have unique electrical and mechanical properties. For example, CNTs have high stiffness with a Young’s modulus of approx. 1 TPa [1, 2], which makes them suitable for applications such as structural materials for cars and space ships. The toughness of CNTs has attracted interest in the field of orthopedics for their use as biomaterials for bone regeneration [3, 4].

Mesenchymal stem cells (MSCs) have the potentials to self-renew and differentiate into the multilineage phenotypes derived from mesoderm, *e*.*g*., osteoblasts, chondrocytes and adipocytes [5, 6]. During osteogenic differentiation, MSCs differentiate into pre-osteoblasts, which become mature osteoblast and later on osteocytes [7]. Furthermore, MSCs has been originally isolated from the bone marrow, but it was reported over the last decades that they can be isolated from the adipose tissue, umbilical cord blood, placenta, dental pulp, and many other adult tissues, and thus are an attractive cell source for tissue engineering that supports autologous cell-based therapies in the field of regenerative medicine [6, 8–11]. Strategies to control proliferation and differentiation of MSCs and direct their fate are present challenges in tissue engineering. In recent years, some biophysical factors such as material stiffness as well as biochemical factors that can affect MSC behaviors (proliferation, differentiation and migration) have been reported [12–15]. Understanding their factors and the extracellular environment that provides them is essential to create new technologies that control the growth and differentiation of MSCs.

CNTs have a cylindrical nanostructure consisting of carbon atoms, and are classified as single-walled (SWNT) or multi-walled nanotubes (MWNT). While CNTs have excellent mechanical properties, their low dispersity in water derived from their hydrophobic character gives rise to intractable disadvantages. Although detergents and alcohols are generally applied to disperse CNTs, the density limitation for the dispersion is not very high [16–18]. To get high dispersity, CNTs conjugated with hydrophilic functional groups and molecules have been developed [19–21]. However the water dispersed CNTs are cytotoxic to human mesenchymal stem cells (MSCs) [22, 23], and the surface with densely immobilized CNTs also reported to be less biocompatible [24]. The functionalized CNTs immobilized on a base material may exfoliate and resuspend in the medium.

4-*O*-methyl-α-d-glucuronoxylan (GX) solution is a remarkable dispersant of CNTs and enables us to disperse them at high concentration without chemical modification [25,26]. The dispersion method using GX is a very attractive for high-density intact CNT coating and film preparation. In the following, we describe the preparation of glass densely coated with intact CNTs using GX solution and their application as substrates to stimulate MSC incubated on these surfaces. The aim of this study is to examine the effect of a surface coating with high-density CNTs on cell adhesion and the osteogenic differentiation of rat MSCs.

## Materials and methods

### Preparation of CNT-coated glass

Single-walled CNTs (SWNTs) were provided by Unidym (Sunnyvale, CA) and multi-walled CNTs (MWNTs) were provided by Nitta (Osaka, Japan). SWNTs and MWNTs were dispersed in 0.2% (w/v) GX solution (IPE Inc., Sakai, Japan) at 0.4 mg/ml. CNT-dispersed solutions (100 μl) were dropped and dried onto 12-mm glass disks (Matsunami, Kishiwada, Japan). After washing in distilled water a few times, the CNT-coated glass disks were dried and sterilized at 180°C. When glass disks are employed as cell culture substrates, poly-l-ornithine (PLO; Sigma-Aldrich, St. Louis, MO) coating is often used to promote cell adhesion to the glass surface [27]. Not only uncoated glass disks but also glass disks coated with PLO were prepared as controls.

### Characterization of CNT-coated glass

#### Atomic force microscopy (AFM)

The uncoated glass and CNT-coated glass surfaces were characterized by atomic force microscopy. The AFM topographs were recorded employing a NanoScope IIIa (Bruker) upgraded to version 8, and equipped with a J-scanner, nominal maximum scanning area of 125 μm x 125 μm. The height topographs were determined in Scan Asyst mode employing a Scan Asyst Air cantilever with nominal spring constant 0.4 N/m. The samples were characterized using scan areas between 1 μm x1 μm to 30 μm x 30 μm, and captured at 512x512 datapoints. The height topographs were flattened employing a quadratic background tilt.

### Cell culture

Primary cultures of rat MSCs were prepared from the femora of 7 week-old male Fisher 344 rats (Japan SLC, Hamamatsu, Japan), as previously reported [28, 29]. The animals were sacrificed following the protocol approved by the ethical committee of animal experiments in Osaka Prefecture University. The MSCs were expanded in 10 cm culture dishes in the presence of Eagle’s minimum essential medium (MEM, Sigma-Aldrich) with 15% (v/v) fetal bovine serum (FBS) and antibiotic-antimycotic solution (100 unit/ml penicillin, 100 unit/ml streptomycin and 250 ng/ml amphotericin B, Nacalai Tesque, Kyoto, Japan). The cell cultures were incubated in an atmosphere including 5% (v/v) CO_2_ at 37°C. The culture medium was changed every few days. After reaching confluence, MSCs were trypsinized using 0.05% (w/v) trypsin/EDTA solution (Nacalai Tesque), and a cell suspension was prepared to achieve a cellular concentration of 50,000 cells/ml. To induce osteogenic differentiation, MSCs were suspended in culture medium supplemented with 10 mM β-glycerophosphate disodium salt (FUJIFILM Wako Pure Chemical, Osaka, Japan), 10 nM dexamethasone (FUJIFILM Wako Pure Chemical) and 82 μg/ml L-ascorbate phosphoester (FUJIFILM Wako Pure Chemical), and then 50,000 cells were seeded in each well of a 24-well plate, one CNT-coated or control glass per well. The cell cultures for osteogenic induction were maintained for 4 weeks with medium replacement every few days.

### Cytochemical staining

#### Immunocytochemistry

Cells cultured for 7 days were fixed in a phosphate buffered saline (PBS) containing 4% (w/v) paraformaldehyde (PFA) for 30 minutes at 4 °C. After removal of PFA solution and washed with PBS, the cells were permeabilized in PBS containing 0.3% (v/v) Triton X-100 solution, washed in PBS and incubated in PBS containing 10% (v/v) goat serum and 0.01% (v/v) Triton X-100 for 1 h at room temperature. Next, the cells were reacted overnight with anti-vinculin mouse monoclonal antibodies (1:200; Sigma-Aldrich) in PBS containing 10% (v/v) goat serum at 4 °C. Following washes in PBS, the cells were reacted with anti-mouse IgG antibodies conjugated with FITC (1:500; Invitrogen, Carlsbad, CA), rhodamine-labeled phalloidin (Cytoskeleton, Denver, CO) and DAPI in PBS containing 10% (v/v) goat serum for 1 h at room temperature. After three washes in PBS, the cells were mounted on slide glasses with PermaFluor mounting medium (Thermo Electron, Pittsburgh, PA).

#### Alkaline phosphatase (ALP) staining

ALP staining was applied to MSCs undergoing osteogenic differentiation to visualize the presence of pre-osteoblasts and osteoblasts [30]. Cells were cultured for 3, 7, 14, 21 or 28 days at 37 °C under 5% CO_2_ in a 24-well plate, and then were fixed by incubating with 1 ml/well of the 4% (w/v) PFA for 30 min at 4 °C. The fixed cells were washed with PBS and incubated with 1 ml/well of the ALP staining solution (0.2 M Tris HCl, pH 9.0, *N*,*N*-dimethylformamide, 0.3 mg/ml naphthol AS phosphate, and 0.6 mg/ml Fast Blue BB salt) for 30 min in darkness at 37 °C. After the solution was removed, the sample was washed again with PBS.

#### Alizarin red S staining

Alizarin red S staining was applied to visualize the deposition of calcium phosphate. Cells were fixed in the same manner as for ALP staining described above. The fixed cells were washed again with PBS and incubated with 1 ml/well of Alizarin red staining solution (0.5% (w/v) Alizarin Red S (Sigma-Aldrich) in PBS for 4 min at 37 °C. After the solution was removed, the sample was washed again with PBS.

### Determination of biochemical markers

MSCs were collected from cultures on the CNT-coated glasses in one 24-well plate at 3 h, 3, 7, 14, 21 and 28 days in a manner similar to that previously described for ALP staining. After the cells were washed twice with PBS, 0.5 ml of 0.2% (v/v) Triton X-100 was added to each well. The plate was incubated for 30 min on ice. Cells were mechanically scraped off using a cell scraper (Sumitomo Bakelite, Tokyo, Japan). The detached cells were triturated with a pipette, collected in a 1.5 ml microtube, mixed vigorously with a Vortex mixer, and used as a sample solution for the measurement of ALP activity, DNA content and calcium content. ALP activity was measured using a LabAssay^™^ ALP Kit (FUJIFILM Wako Pure Chemical) according to the manufacturer’s specifications. Briefly, a sample solution (20 μl/well) was added to the pre-warmed substrate solution (100 μl/well) in a 96-well microplate and was incubated for 15 min at 37 °C to measure the ALP activity. The substrate solution contained carbonate buffer (pH 9.8), 2 mM magnesium chloride and 6.7 mM sodium *p*-nitrophenylphosphate hexahydrate (*p*NPP). After the addition of the 0.2 M NaOH (80 μl/well) to stop the enzymatic reaction, absorbance at 405 nm was measured. *p*-Nitrophenol (*p*NP) was used as a calibration standard. The value of the DNA content in each sample was used to standardize the value of each biochemical marker per cell according to the previous reports [29–31]. A sample solution (200 μl) was mixed with 1.8 ml TEN buffer (10 mM Tris, 1 mM Na_2_EDTA, 0.1M NaCl, pH 7.4) and the DNA content was measured using the Hoechst 33342 solution (Dojindo Laboratories, Mashikimachi, Japan). The fluorescence intensity (Ex 350 nm, Em 461 nm) of the sample were determined employing a fluorescence spectrophotometer (RF-5000, Shimadzu, Kyoto, Japan).

### Inductively-coupled plasma emission spectrometry (ICPES)

The calcium content were determined by mixing an aliquot (500 μl) of the sample with concentrated hydrochloric acid (500 μl) in a glass vial container and incubated at room temperature overnight. The sample was subsequently diluted 50-fold and the calcium content was determined employing a inductively-coupled plasma emission spectrophotometer (Optima 4300, Perkin Elmer, Waltham, MA). Calibration standard solution of calcium (37509–04 Nacalai Tesque) was used.

### Gene expression analysis

Total RNA was extracted from MSCs with an RNAqueous kit (Ambion, Austin, TX) according to the manufacturer’s specifications. Complementary DNA was synthesized with a PrimeScript RT reagent kit (Takara Bio, Kusatsu, Japan) for reverse transcription (RT) using oligo-dT primers and random 6-mers. Polymerase chain reaction (PCR) was performed with SYBR Premix Ex Taq II (Takara Bio) on a real-time PCR thermal cycler Opticon (Bio-Rad Laboratories, Hercules, CA) with gene-specific primers for alkaline phosphatase (*Alpl*), bone morphogenic protein 2 (*Bmp2*), osteocalcin (*Bone gamma-carboxyglutamate protein*: *Bglap*), runt-related transcription factor 2 (*Runx2*), and glyceraldehyde-3-phosphate dehydrogenase (*Gapdh*). The PCR protocols were as follows: 95°C for 30 s, then 40 cycles of 95°C for 5 s, and 60°C for 30 s. The primer sequences are shown in Table 1. The comparative delta delta cycle time (ΔΔC_T_) method was used to quantify gene expression levels, following the protocol of the manufacturer. The levels of target gene expression were normalized to the level of *Gapdh* expression.

**Table 1:** Primer sequences for quantitative RT-PCR.

Target	Sequences (5′-3′)	Tm
*Alpl*	Forward: ACCTGACTGACCCTTCCCTCT	65.2
	Reverse: TGCTTGGCCTTGCCTTC	65.3
*Bmp2*	Forward: ACCGTGCTCAGCTTCCATC	65.5
	Reverse: GAAGAAGAAGCGTCGGGAAGT	65.7
*Bglap*	Forward: CCTCTCTCTGCTCACTCTGCTG	65.8
	Reverse: CCTTACTGCCCTCCTGCTTG	65.9
*Runx2*	Forward: TACGAAATGCCTCTGCTGTTATG	64.9
	Reverse: CTTGGGGAGGATTTGTGAAGA	65.0
*Gapdh*	Forward: TGCATCCTGCACCACCA	66.8
	Reverse: TCACGCCACAGCTTTCCA	67.0

### Western blotting

MSCs were homogenized using ice-cold RIPA buffer (Thermo Scientific, Rockford, IL) containing protease inhibitor cocktail (Nacalai Tesque), followed by centrifugation at 14,000 g for 15 min at 4°C. The supernatant were collected as the protein samples, and then their concentrations were determined using Protein Assay Bicinchoninate kit (Nacalai Tesque). Protein samples (6 or 11 μg) were incubated at 90°C for 3 min with Sample Buffer Solution (6×) (Nacalai Tesque), and then separated by 12.5% sodium dodecyl sulfate-polyacrylamide gel electrophoresis (SDS-PAGE) and transferred onto PVDF membranes (Atto, Tokyo, Japan). Membranes were blocked in 0.1% bovine serum albumin (BSA) at room temperature for 1 h, and then incubated with the first antibodies diluted in PBS-Tween (0.137 M NaCl, 2.7 mM KCl, 8.1 mM Na_2_HPO_4_, 1.5 mM KH_2_PO_4_, and 0.1% Tween-20) containing 0.1% BSA at 4°C overnight. The first antibodies were anti-β-actin (Applied Biological Materials, Richmond, BC, Canada), anti-Runx2 (MBL, Nagoya, Japan), anti-Osteocalcin (Cosmo Bio, Tokyo, Japan), and anti-Osteopontin (Rockland, Limerick, PA). The membranes were washed and incubated with the second antibodies diluted in PBS-Tween containing 0.1% BSA at room temperature for 1 h. The second antibodies were horseradish peroxidase-conjugated anti-mouse IgG (Millipore, Temecula, CA) and anti-rabbit IgG (Millipore). The bands were detected with the ImmunoStar Zeta chemiluminescence detection regent (FUJIFILM Wako Pure Chemical) according to the manufacturer’s specifications. Image acquisition was performed by the imaging system Ez-Capture MG (Atto).

### Scanning electron microscopy

MSCs were grown as described above and fixed at day 28. After washing in double distilled water three times, the samples were dried at 60°C for 1 day. The mineralized parts of the samples were observed using a scanning electron microscope (SEM), the Energy Dispersive X-ray Spectroscopy (EDX) system (SU1510, Hitachi High-technologies, Tokyo, Japan).

### Statistical analysis

All data are represented as the mean ± SD. Single factor analysis of variance (ANOVA) tests and post-hoc tests were used to determine statistical significance.

## Results

### Characterization of surfaces coated with CNTs

**Fig 1:**
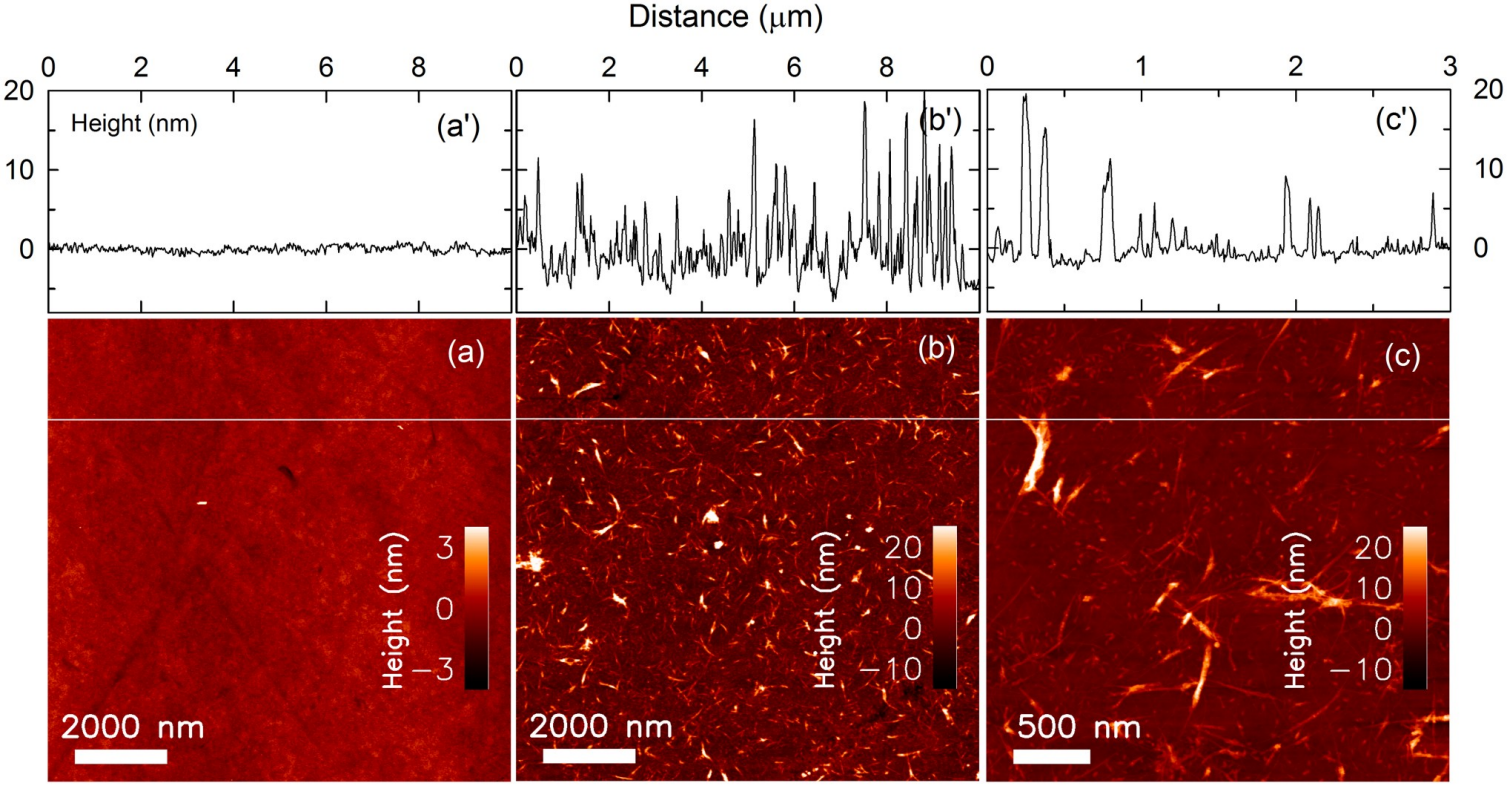
AFM topographs with cross sections of the uncoated glass and the SWNT coated glass. AFM height topographs of (a) the uncoated glass, (b) the SWNT densely coated glass and (c) the SWNT thin coated glass, and a’, b’ and c’ are the height information of cross-section view along the white lines shown on the topographs a, b and c, respectively.

Glass specimens were densely coated using 0.4 mg/ml CNTs dispersion in GX solution and dried. The AFM topography of the SWNT coated glass showed elongated, stiff structures oriented in various directions (Fig 1). The SWNT structures had a height up to 15–20 nm over the uncoated glass (Fig 1b’ and 1c’) clearly distinguishable from the height variations of less than a nm for the uncoated glass (Fig 1a’). The thinner SWNT structures were observed with a height of 3–5 nm (Fig 1c’) being significantly less than the more bundled, thicker structures. These thinner SWNT structures appeared almost rectilinear in the AFM topographs with length up tp 500 nm (Fig 1b and 1c), indicating a significant stiffer nature than for DNA reported to a persistence length of 50 nm. The roughness of the SWNT coated glass of 4.7 nm was significant larger than the value of 0.5 nm determined for the uncoated glass.

### Cell adhesion on CNT-coated glass

**Fig 2:**
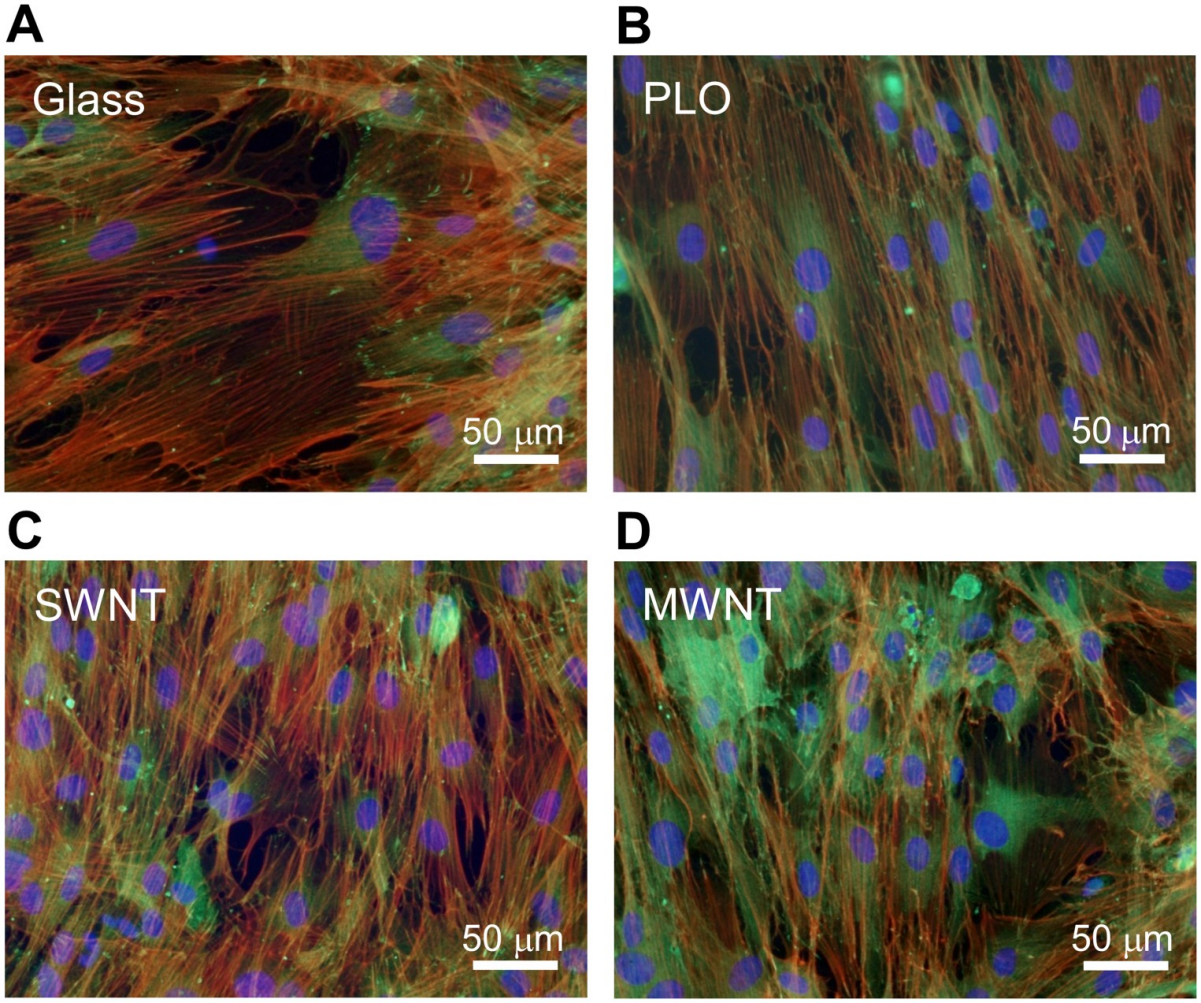
Fluorescent images of MSCs with stained cytoskeleton and focal adhesion. Cells were stained with DAPI (blue, nuclei), rhodamine-phalloidin (red, actin), and anti vinculin antibodies (green, vinculin), spread widely with a lot of actin fibers and focal adhesions on (A) uncoated glass, (B) PLO-coated glass, (C) SWNT-coated glass, and (D) MWNT-coated glass.

Immunocytochemical analysis was performed on the cells maintained in the culture medium on each glass for 7 days. We found that there was no detectable difference in cell morphology on CNT-coated glass comparing to uncoated and PLO-coated glass as controls. The analysis shows that adherent and spread MSCs on CNT-coated glass possessed numerous anchoring points by focal adhesion and definite stress fibers consisting of F-actin (Fig 2).

### Expression of osteogenic genes and proteins in MSCs

**Fig 3:**
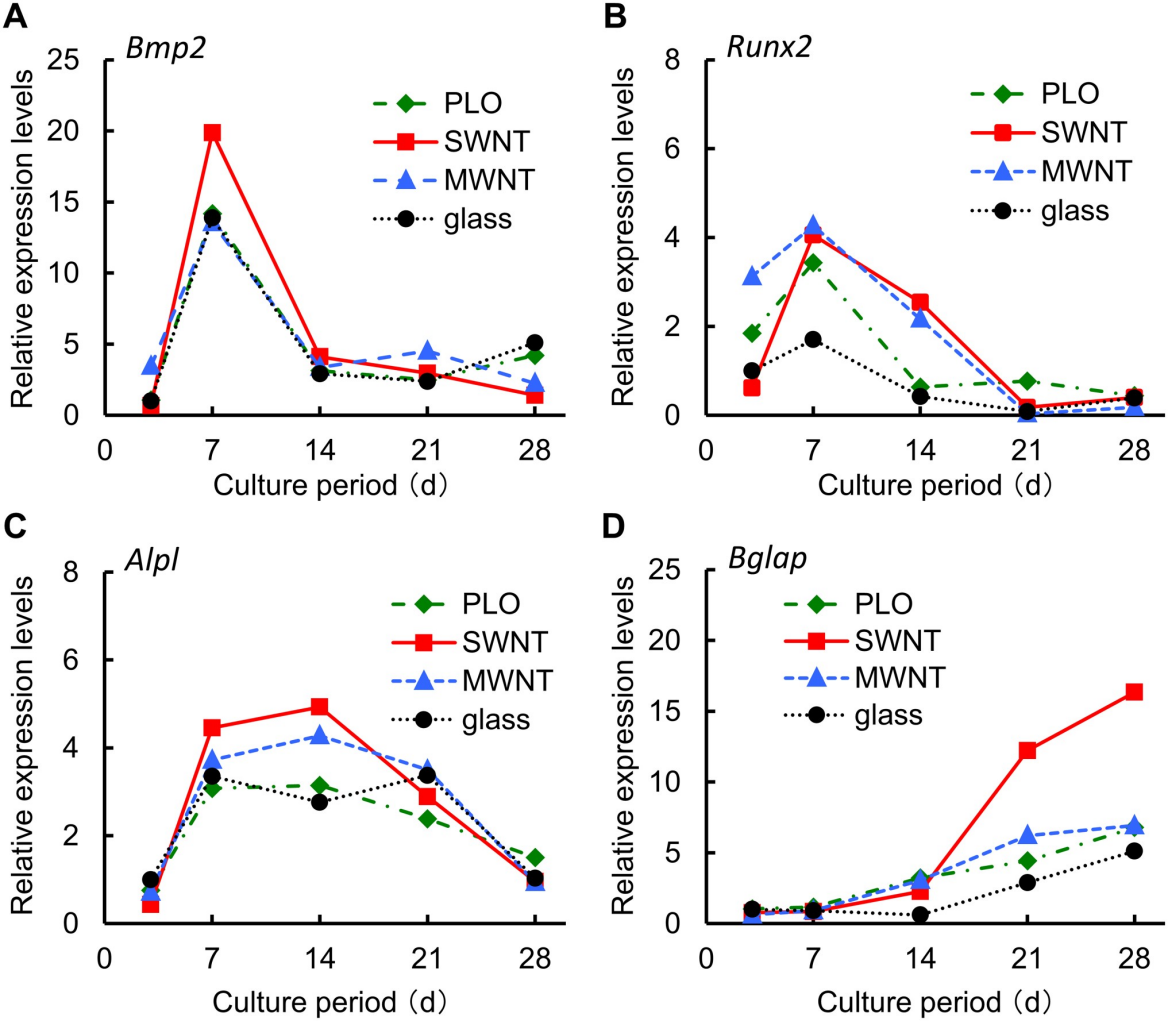
Expression analysis of the genes associated with osteogenic differentiation. The expression levels of (A) *bone morphogenic protein 2* (*Bmp2*), (B) *runt-related transcription factor 2* (*Runx2*), (C) *alkaline phosphatase* (*Alpl*), and (D) *osteocalcin* (*Bglap*) for MSC incubated on uncoated glass, and glass coated with SWNT, MWNT and PLO. Results are means of the duplicate experiments relative to the expression levels for MSC grown on uncoated glass at day 3.

We evaluated the expression levels of *Bmp2*, *Runx2*, *Alpl*, and *osteocalcin* (*Bglap*) to analyze the difference in expression patterns of osteogenic genes under differentiation-inducing culture conditions. The expression levels of *Bmp2*, which reflects enhanced bone morphogenesis, peaked at day 7 (Fig 3A). The peak in this expression level for cells cultured on SWNT coated glass was particularly high, but the trend in the expression levels was the same for cells incubated on the three coated glasses. The expression levels of *Runx2*, a transcriptional factor accelerating osteogenesis, peaked at day 7. The peak was highest for the SWNT- and MWNT-coated surfaces, and the expression levels remained higher than uncoated and PLO-coated glass until day 21 (Fig 3B). The expression pattern of *Alpl* peaked at day7 or day14 for each type of surface coating, with the highest peaks in the expression levels were observed for incubation on the SWNT-coated glass whereas uncoated and PLO-coated glass yielded an expression level of about two-thirds of that observed for incubation on the SWNT coated glass (Fig 3C). Osteocalcin is implicated in bone mineralization and is used as a marker for osteoblasts [32]. The *Bglap* expression levels in every sample gradually increased after day 14 and the samples of day 28 showed the maximum value during osteogenic culture. MSCs on SWNTs were the highest value in the experiment (Fig 3D).

**Fig 4:**
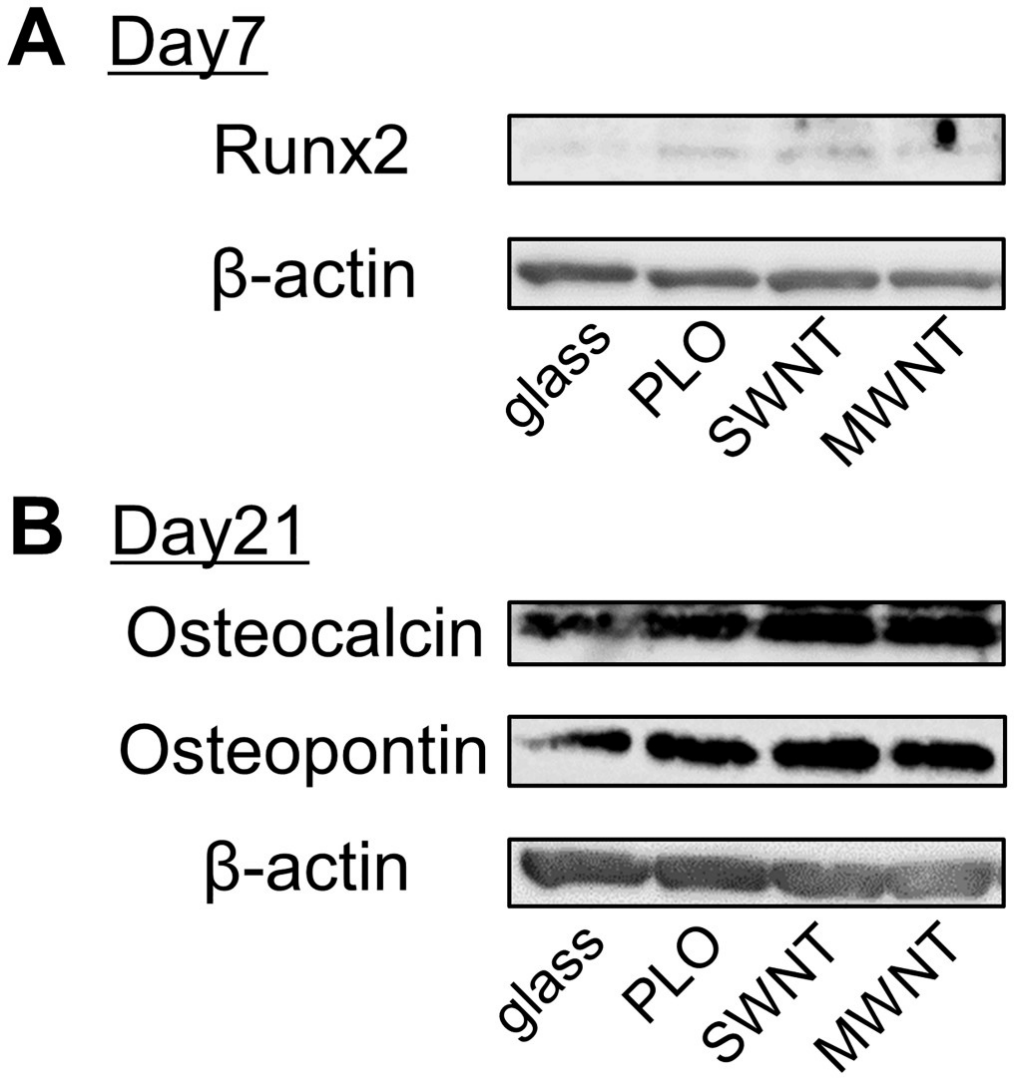
Expression of the proteins associated with osteogenic differentiation. Protein samples were made from MSCs incubated on uncoated glass, and glass coated with SWNT, MWNT and PLO for 7 days. The bands show the expressions of (A) Runx2 in the 6 μg of protein samples obtained from MSCs incubated for 7 days, and (B) Osteocalcin and Osteopontin in the 11 μg of protein samples obtained from MSCs incubated for 21 days.

Western blotting experiments confirm the upregulation of Runx2 proteins in MSCs incubated on SWNT-, MWNT- and PLO-coated glass at day 7 (Fig 4A). Additionally, the high expressions of osteogenic marker proteins, osteocalcin and osteopontin (secreted phosphoprotein 1: Spp1) were shown in MSCs incubated on SWNT- and MWNT-coated glass at day 21 (Fig 4B).

### ALP activity of MSCs during osteogenic induction

**Fig 5:**
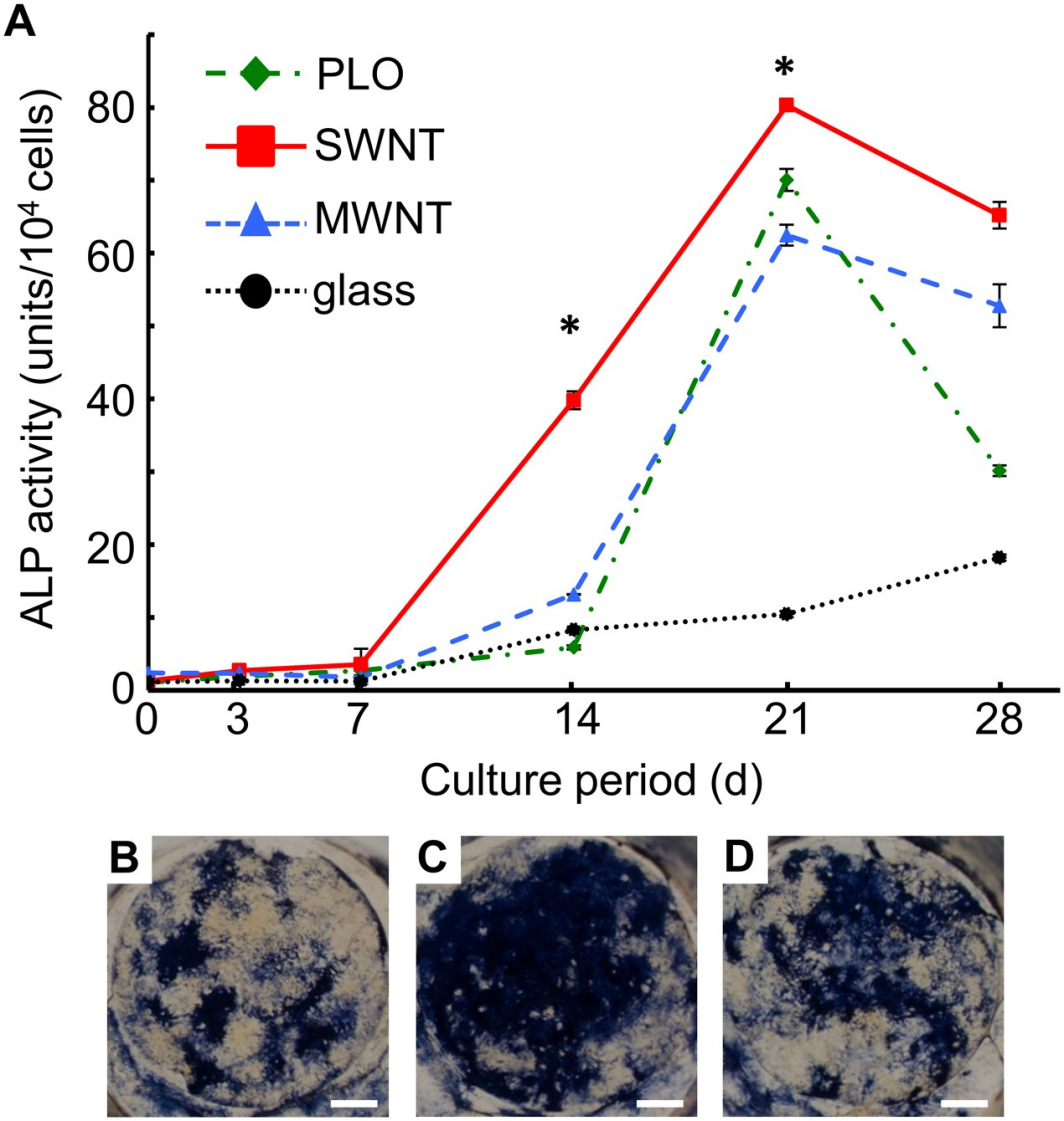
ALP activity of MSCs during osteogenic differentiation. Cells were cultured in osteogenic differentiation medium for 28 days. (A) ALP activities normalized by cell numbers were determined day 3, 7, 14, 21, and 28. The data are presented as mean ± S.D., n = 6 (*: p<0.05). In addition, ALP-stained cells are shown on (B) PLO-, (C) SWNT-, (D) MWNT-coated glass at day 28. Scale bar: 2 mm.

ALP activity is used to estimate the amount of differentiated pre-osteoblasts and osteoblasts for each sample. ALP activity increased rapidly from day 7 after osteogenic induction, and peaked at day 21 for all surfaces except for uncoated glass. ALP activity was highest for the SWNT-coated glass (Fig 5A). ALP staining shows the distribution of pre-osteoblasts and osteoblasts differentiated from MSCs on uncoated or the three coated glass surfaces (Fig 5B–5D).

### Mineralization of MSCs

**Fig 6:**
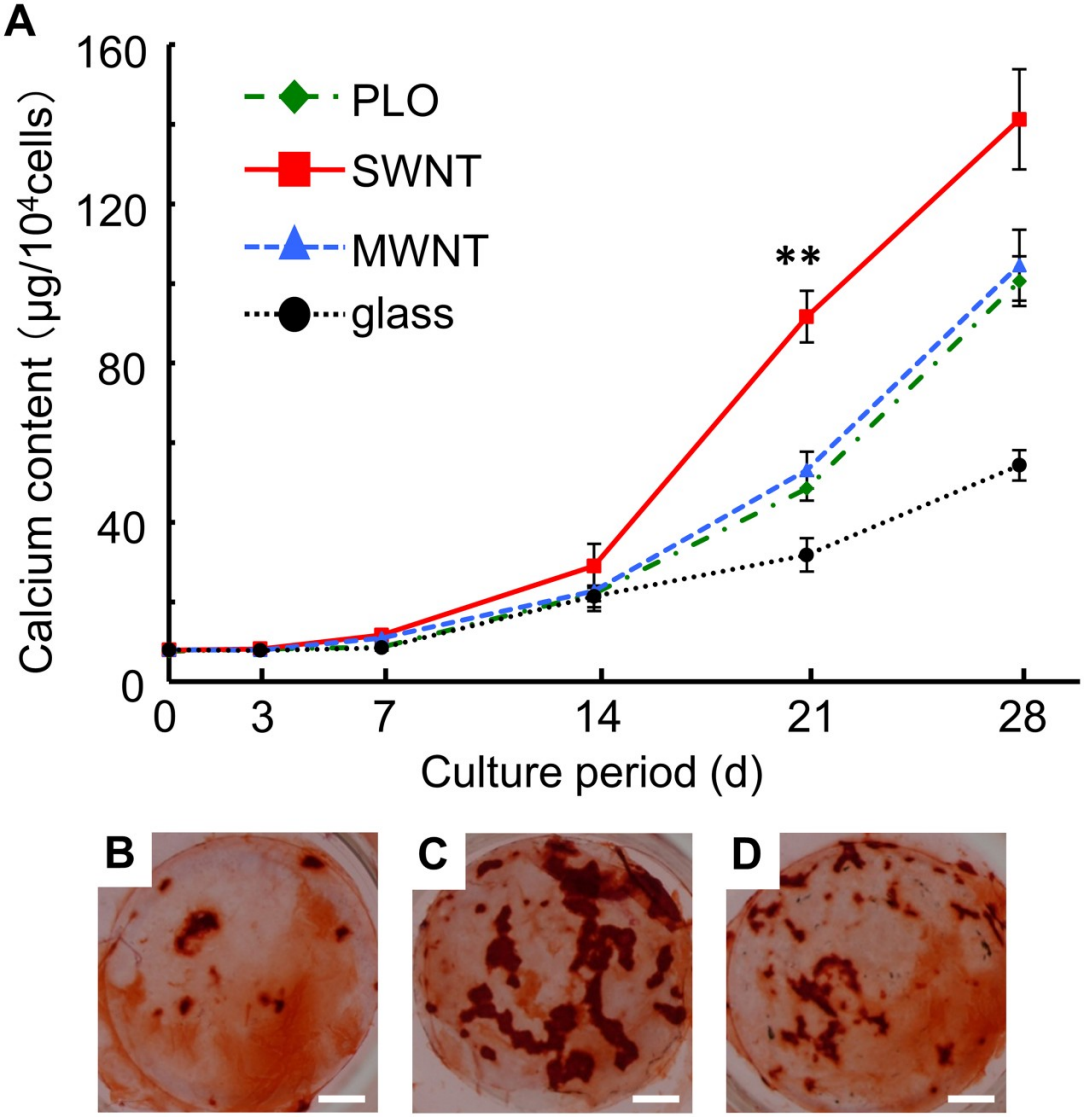
Calcium phosphate deposited by MSCs during osteogenic differentiation. Cells were cultured in osteogenic differentiation medium for 28 days. (A) Calcium contents normalized by cell numbers were measured at day 3, 7, 14, 21, and 28. The data are presented as mean ± S.D., n = 6 (**: p<0.01). In addition, alizarin red-stained cells are shown on (B) PLO-, (C) SWNT-, (D) MWNT-coated glass at day 28. Scale bar: 2 mm.

In order to examine bone mineralization after osteogenic differentiation, we stained using alizarin red S and measured calcium deposits using ICP. The amount of calcium increased over the course of the culture period, and the value for the SWNT sample was almost three times higher than that of the glass sample on day 28 (Fig 6A). The PLO and MWNT samples were virtually identical, and were about 70% of the SWNT value. The SWNT sample showed strong staining by alizarin red S comparing to the PLO and SWNT ones on induction day 28 (Fig 6B–6D).

**Fig 7:**
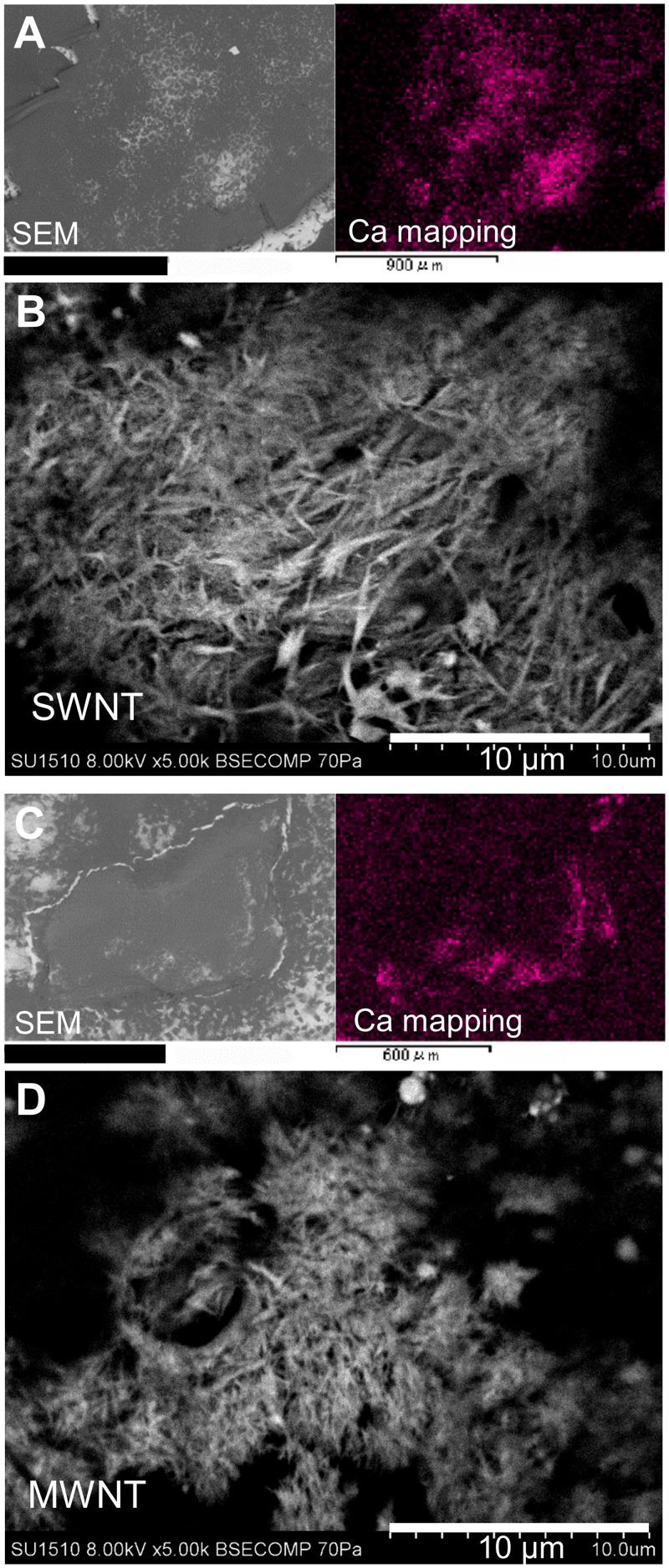
SEM-EDX analysis of calcium phosphate deposited by MSCs on SWNTs and MWNTs. The samples were observed by SEM and the localization of calcium was analyzed on (A, B) SWNTs and (C, D) MWNTs by the EDX system. A and C are low magnification images using SEM (left) and calcium mapping of the same view (right). B and D are high magnification images using SEM. Scale bar: (A) 900 μm, (C) 600 μm.

To determine the state of calcium phosphate crystals formed in the SWNT and MWNT, samples, we performed calcium mapping on day 28 using a SEM-EDX system (Fig 7A and 7C). The crystals in both samples had a needle-shaped hydroxylapatite-like crystal form (Fig 7B and 7D).

## Discussion

Here we have shown that glass surfaces coated with SWNTs at high concentrations are able to facilitate the osteogenic induction of rat mesenchymal stem cells (MSCs) better than PLO- (as control) and MWNT-coated glass as well as uncoated control. Mooney *et al*. have reported the proliferation and differentiation of MSCs on SWNTs and MWNTs [24]. However, their results showed decreasing cell viability at high concentrations of COOH-functionalized SWNT (not less 0.16 mg/ml) and OH-functionalized MWNT (not less 0.032 mg/ml), and they did not address whether CNTs act as osteogenic enhancers or not [24]. While improvement of CNT aqueous dispersion readily can be accomplished by CNT functionalization, the resulting dispersed CNTs may at the same time inhibit cell proliferation. Liu *et al*. have reported that dispersed carboxylated CNTs inhibit the proliferation and differentiation of MSCs [22]. Usui *et al*. showed high bone-tissue compatibility *in vivo* by injection of MWNTs with collagen and BMP2 [33]. Here, we demonstrate stable cell adhesion and the promotion of osteogenic differentiation of MSCs on SWNTs (Figs 2 and 3). This strategy is based on the application of a dispersant for the SWNT and MWNT in the process of surface coating glass. Some detergents, alcohols, nucleotides, and polysaccharides have been developed as dispersants for CNTs, though they have problems with cytotoxicity and limitation on dispersible amounts [16–18]. Here, we achieved the high density of CNTs on glass disks using a polysaccharide dispersant, GX solution (Fig 1). The density of CNTs (approx. 35 μg/cm^2^) was beyond the level recently reported as the CNT film prepared for osteoblast culture [34]. The present result suggested CNTs were forming an interconnected mesh on the surface with height variations in the range up to 20 nm (Fig 1) which is supported by the percolation theory [35]. In many case of CNT application for tissue engineering previously reported, CNT can be coated on the surface of other materials including e.g., polystyrene culture dish, polymer nanofibers, ceramics, polymer hydrogels and sponges. It was reported that “compact” with very high density of CNT induced osteogenic differentiation of human MSCs [36]. Thus, the osteogenic induction of MSCs found here emerge from a higher density of CNT on the glass surfaces.

Osteogenesis can be explained as the process in which MSCs differentiate into pre-osteoblasts, which further give rise to mature osteoblasts specialized for the secretion of extracellular matrix (ECM) and mineralization [7]. Transcriptional factor Runx2 is essential for the commitment of MSCs to osteoblast lineages, induces osteoblast differentiation with upregulation of bone matrix genes expression (*Col1a1*, *Spp1*, *Bglap*, etc.) and increases the number of immature osteoblasts during bone development [37, 38]. BMP2 activates osteogenic differentiation of MSCs through the Smad-dependent BMP signaling pathway, and is responsible for differentiation [39, 40]. ALP has a role in removing phosphate groups from many kinds of molecules, including nucleotides and proteins. During osteoblast differentiation, ALP is an important stimulator of bone formation and is a marker to detect cells forming pre-osteoblasts and early osteoblasts [29, 30, 41]. *Osteocalcin*/*Bglap* is a gene for osteoblast differentiation and mineralization depending on the maturation, and is expressed in the late stages [38, 42]. In this study, the expression levels of *Runx2* and *Alpl* were high in the first two weeks in differentiating cells on SWNT-coated and MWNT-coated glass (Fig 3B and 3C). These results suggest that both CNT-coated glasses promote early osteoblast differentiation in MSCs. But in late stage osteoblast differentiation only the SWNT-coated glass showed higher expression of *osteocalcin* and mineralization than the PLO-coated and uncoated control. Finally, calcium phosphate deposited on the SWNT-coated glass had a thicker and longer apatite-like needle shape than that on MWNT-coated glass. SWNTs seem to provide a better environment suitable for mineralization and deposition, compared with MWNTs in late stage bone formation.

Which factors affected the difference in osteogenic induction of MSCs between the CNT-coated glass disks and controls (PLO-coated and uncoated glass disks)? We think there are several possible mechanisms. Surface nanotopography of culture substrates affects stem cell behaviors including cell proliferation and differentiation [12, 43]. MSCs would response to the differences of roughness generated by CNTs. We need to investigate the relationship between the surface roughness of CNT-coated glasses and the differentiation of MSCs in the future work. Another factor possibly affecting the induction of MSCs are difference in adsorption of ECMs onto the glass surface because of the difference in hydrophobicity.

## Conclusions

Our results show that densely SWNT-coated glass promotes osteogenic differentiation and mineralization of rat bone marrow mesenchymal stem cells *in vitro*. In contrast, MWNT-coated glass did not promote mineralization in spite of high expression of *Runx2* that is a transcriptional factor of early osteogenesis. This study suggests that SWNT coating at high density may lead to a new approach for bone regeneration.
